# Effect of Xinjiang Uyghur* Vernonia anthelmintica* Willd Injection Treatment with Silicosis Fibrosis

**DOI:** 10.1155/2016/5139651

**Published:** 2016-10-10

**Authors:** Guitao Liu, Yingjia Liu, Lu Jin, Cuidong Li, Liping Nie, Yanhua Wei, Wenyu Wang, Yuhong Xu, Nuziguli Nusilaiti, Ping Hua, Xiujuan Li, Xiaohua Wang, Wenlong Wei, Hamulati Wufuer

**Affiliations:** ^1^Department of Occupational Disease Prevention and Control, The Fifth Affiliated Hospital, Xinjiang Medical University, Urumqi, Xinjiang 830011, China; ^2^Xinjiang Medical University, Urumqi, Xinjiang 830054, China; ^3^Department of Pathology, The Fifth Affiliated Hospital, Xinjiang Medical University, Urumqi, Xinjiang 830011, China

## Abstract

*Objective*. To observe the curative effect of VAWI on Xinjiang Uygur patients with silicosis fibrosis.* Methods*. After we diagnosed the 40 patients with the first phase of silicosis, we randomly divided them into two groups: the basic treatment group (group A, *n* = 20) and the VAWI group (group B, *n* = 20). At the same time, we selected the age-matched healthy patients (*n* = 20). We applied the combined protein chip with SELDI-TOF-MS to carry out the serum analysis. The data were analyzed throughout data preprocessing, difference in PEAK screening, hierarchical cluster analysis, and Principal Component Analysis (PCA). We built decision tree model and predict the difference between the PEAK corresponding proteins.* Results*. The proteins peaks corresponding to name, predicted protein, and gene name were as follows: M2001_69, amyloid beta a4 protein, APP, and M2017_02, amyloid beta a4 protein, APP. The different expression of proteins in patients with silicosis was found before and after with VAWI treatment. The predicted proteins were as follows: M1982_50, amyloid beta a4 protein, APP; M3164_50, fibrinogen alpha chain frag, FGA; M3379_28, fibrinogen alpha chain frag, FGA; and so on.* Conclusion*. VAWI presented curative effect on patients with silicosis fibrosis via the alternation of proteins expression in serum.

## 1. Introduction

The mechanism of pneumoconiosis is a long-term inhalation of productive dust which leads to the pulmonary fibrosis. It is one of the most serious occupational disease hazards in China. Pneumoconiosis has 13 types including silicosis and coal workers pneumoconiosis [1, 2]. The treatment of patients with silicosis fibrosis remains to be improved; the study of diagnosis and treatment of silicosis thus should be launched.

Proteomics technology includes purification technology and protein identification technology. Mass spectrometry technology is the core of the protein identification technology [3]. The surface enhanced laser desorption ionization time of flight mass spectrometry (SELDI-TOF-MS) is a kind of ultramicro, high flux, fully automatic screening protein technology which can detect a variety of samples including serum, urine, and cell [4].

This research utilized the Xinjiang specialty drug,* Vernonia anthelmintica* Willd Injection (VAWI), for the treatment of patients with silicosis, and conducted detection of SELDI-TOF-MS to observe the curative effect on silicosis fibrosis patients treated with VAWI. We aim to study the effect of VAWI treatment on patients with silicosis fibrosis and provide a future basis for the rational development of novel therapy.

## 2. Materials and Methods

### 2.1. Clinical Methods

#### 2.1.1. Selected Clinical Cases of Grouping Method

According to the inclusion criteria and imaging technology of 600 cases of silicosis patients, we selected 40 cases. We divided these into two groups, A-Q and B-Q. After conventional treatments and VAWI treatment, we got A-H group and B-H group.

#### 2.1.2. Clinical Treatments

As the basic treatments, the A-H group used penicillin and cephalosporin antibiotics supplemented with cough, phlegm, and asthma common medicine; VAWI group was given basic treatment + VAWI.

#### 2.1.3. Dosing Methods

The course of basic treatment is 15 days including iv fluids of antibiotics and oral drugs of cough expectorant antiasthmatic. 2 mL of VAWI was added with 10 mL of 0.9% saline in the atomization inhalation way, 2 times a day, 10 days to 15 days for a course of treatment.

#### 2.1.4. Diagnostic Criteria

According to GBZ2002 silicosis [5], silicosis diagnosis aptitudes of physician diagnosis, such as reliable SiO_2_ dust exposure history, X-ray radiography as the main basis, reference of clinical manifestation, and laboratory examination were considered, while other similar lung disease, control silicosis diagnosis standards were ruled out [6]. The silicosis patients were diagnosed with stage one, two, or three.

#### 2.1.5. Clinic Information of Silicosis Patients

Silicosis patients conform to silicosis pneumoconiosis diagnosis of the basic standards [7]. The patients enrolled in our study were aged from 60 to 80 years. We only reserved the male cases who cut mountains for railroad from 1950s to 1970s. Pneumoconiosis was found in all patients by chest X-ray detection.

#### 2.1.6. Exclusion Criteria

We selected first phase of pneumoconiosis without coronary heart disease, hypertension, rheumatism, diabetes, liver, or kidney dysfunction.

### 2.2. Serum Samples Information

The study collected 20 clinical serum samples of each group including A-H, A-Q, B-Q, and B-H. These 80 samples were detected through SELDI-TOF-MS (see Table 1).

### 2.3. Equipment Instrument

Ciphergen® SELDI-TOF-MS (surface enhanced laser desorption ionization time of fight mass spectrometry) surface enhanced laser desorption ionization time of flight mass spectrometer (protein fingerprint device) (Northern District, CA, USA) was used in this study.

ProteinChip SELDI system was used to quickly gain protein molecular weight map from a large number of complex biological samples as well as to find biomarkers. Surface enhanced laser desorption ion technology was used to capture, detect, and measure the molecular weight of peptides and proteins in complex biological samples [8].

### 2.4. Experiment Method

The use of SELDI protein chip includes four steps.

#### 2.4.1. Chip Type Selection

The function of the protein chip provides various chromatography, including hydrophilic chromatography, hydrophobic chromatography, cation, anion exchange, and metal bonding surface. In addition, the selected proteins or targeted molecules can preactivate the surface of the chip through covalently coupling, aiming to make the chips have more specificity.

#### 2.4.2. Samples Detection

Serum, cells, or tissues of the cracking fluid, urine, cerebrospinal fluid, or other proteins and serums, complex biological samples—including those samples containing high concentration of salt ions and detergent—can be directly on sample in the protein chip surface. Being on sample can be by manual or automatic instrument way. A particular subgroup of complex protein samples was captured by the chip by simple chemistry or protein interaction.

#### 2.4.3. Uncombined Component Elution

After incubation, uncombined protein and other ingredients from the chip surface cleared off. Only those specific binding proteins are retained for further analysis. This selective elution was further obtained based on the characteristics of protein chip set.

#### 2.4.4. Analysis of SELDI Protein by Reading Machine

After the elution step, the organic solution of energy absorption molecules (EAMs) is added. EAMs played a key role in ionization of the sample. After protein dissolved into a solution containing the EAM, the solution was to volatilize, and it formed in the chip's surface protein and cocrystallization of EAMs.

Chip in SELDI reading machine was analyzed, and the latter was a kind of time of flight mass spectrometry. Chip reading machine was a source of nitrogen laser that causes ionization reconciliation of adsorption process. The laser ionization energy induced protein ionization; then it transformed from crystal to gas.

Once into the gaseous state, proteins molecules were charged under the effect of a separation voltage quick movement, or called “flight”; separation voltage for all the molecules in the sample had the same effect, with difference in time of flight, according to the different molecular weight. SELDI reading machine recorded the time of flight and converted the data into molecular weight.

### 2.5. Contrast Strategy Transform

The following comparison was conducted, respectively, and the data were collected for further bioinformatics analysis:

A-Q versus A-H and B-Q versus B-H for a total of 2 times.

### 2.6. Analysis of the Content

Data analysis included data preprocessing, difference in PEAK screening, hierarchical cluster analysis, PCA, building decision tree model, the difference in the PEAK corresponding protein prediction.

### 2.7. Statistical Methods

Statistical methods were poor application SPSS17.0 software (IBM, Armonk, NY, USA), quantitative data, two sets of equal variance, and using *t*-test; two sets of heterogeneity of variance, with rank and inspection; disorderly classification data, using *χ* ^2^ test; and orderly classification data, with rank and inspection. Alpha = 0.05.

## 3. Results 

### 3.1. Data Preprocessing

Raw data were collected by Ciphergen Protein Chip Software and correction processing, the peak data were also determined, mass-to-charge ratio less than 1000 of the peak is substrate peak, filtered based on the peak conduct subsequent data analysis.

### 3.2. Differences in Peak Filtering

Comparison between samples from different groups was conducted. The peak between two groups was determined by Wilcoxon sum rank statistics test, calculated using *p*-peak value judgment whether or not there were significant differences in the two groups. With 0.05 or 0.01 for *p* value to the value of threshold, at the same time combining OPLS-DA model first principal component of VIP (Variable Importance in the the Projection) values (threshold value > 1), select different peak. Differences between peak screening results were compared: A-Q versus A-H (Table 3) and B-Q versus B-H (Table 4).

### 3.3. The Hierarchical Clustering Analysis

**Figure 1:**
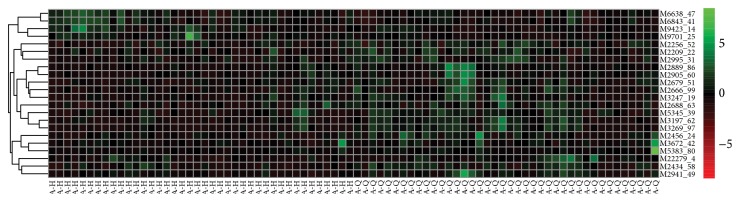
A-Q versus A-H difference in peak clustering results.

**Figure 2:**
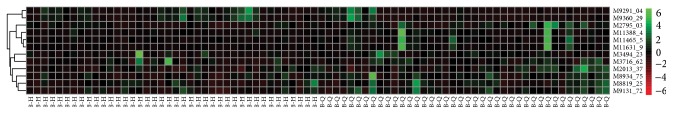
B-Q versus B-H difference in peak clustering results.

Different peaks with Mev software were studied on hierarchical cluster analysis, through the clustering diagram which shows the relationship between the samples. Each line represents a peak in this diagram, each column represents a sample, red shows sample testing content is higher, and green shows sample testing content is low. Each group's compared results of the heat were shown in A-Q versus A-H (Figure 1) and B-Q versus B-H (Figure 2).

### 3.4. PCA

**Figure 3:**
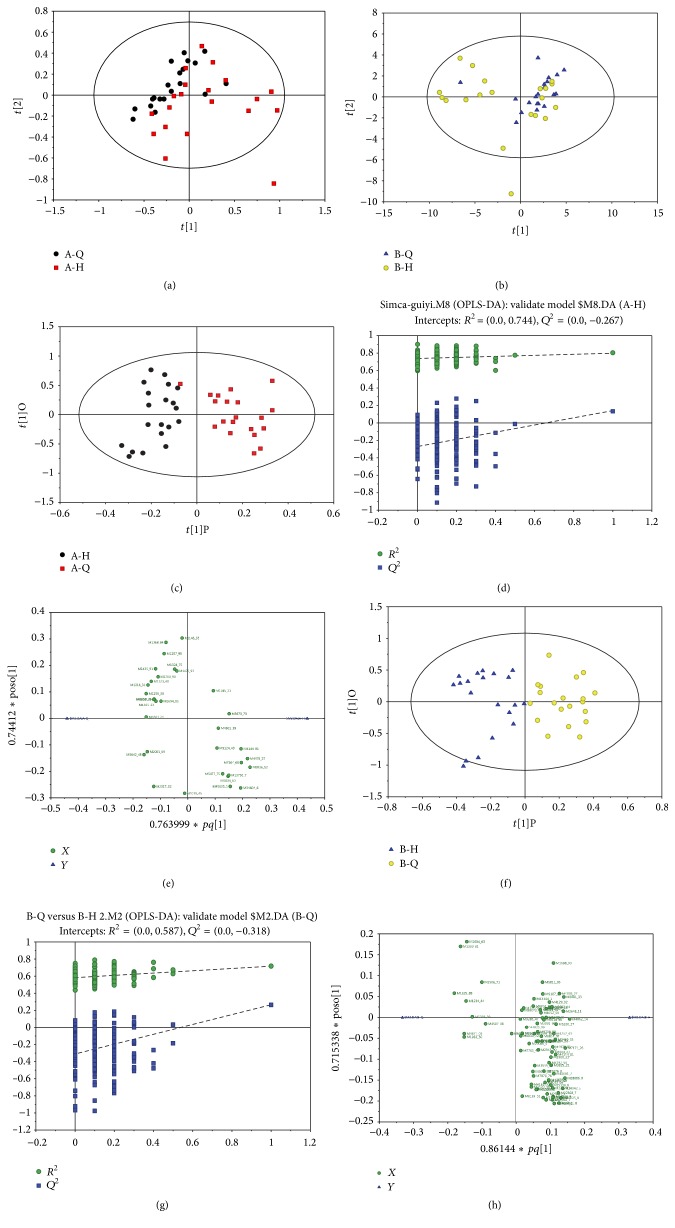
PCA and OPLS-DA results. (a) A-Q versus A-H PCA scoring chart.** (**b) B-Q versus B-H PCA scoring figure. (c) A-Q versus A-H group OPLS-DA scoring figure. (d) A-Q versus A-H group permutation test chart. (e) A-Q versus A-H group OPLS-DA load diagram. (f) B-Q versus B-H group OPLS-DA and score chart. (g) B-Q versus B-H group displacement test diagram. (h) B-Q versus B-H group OPLS-DA load diagram.

According to the principal component analysis, the characteristics of the sample of the amount of compression, in low dimension space, reflect the relationship between the samples. Using SIMCA software (V14, Umetrics AB, Umea, Sweden) the PCA results are shown. The PCA scoring results were exhibited in A-Q versus A-H (Figure 3(a)) and B-Q versus B-H (Figure 3(b)).

### 3.5. OPLS-DA Model Building

Using SIMCA software (V14, Umetrics AB, Umea, Sweden) orthogonal correction of model of the partial least squares discriminant analysis (OPLS-DA) maximizes the highlight model internal and predictive PCA (predictive component) related to the differences. The software used UV scaling for normalization of the data and selected the first principal component and the second principal component to modeling. The quality of the model was analyzed with 7-fold cross-validation test, after using cross-validation of *R* ^2^ *Y* (to represent the interpretability *Y* variable) and *Q* ^2^ (on behalf of the predictability of the model) validity of the model of evaluation. After that, through the arrangement of experiment randomly for many times (*n* = 200) changing the order classification variables *Y* gain random *Q* ^2^ was corresponding to different values of validity of the model for further inspection, including *A* for principal component number and *N* for observation object (sample) number.

Model of accumulation explanation rate was shown in Table 2.

#### 3.5.1. OPLS-DA of Basic Treatments

A-Q group versus A-H group OPLS-DA score plot was shown in Figure 3(c), displacement test (permutation test) diagram was shown in Figure 3(d), and OPLS-DA load diagram was shown in Figure 3(e).

OPLS-DA scoring diagram was shown in Figure 3(c) (abscissa as the first principal component (predicted principal component), expressed in *t*[1]P; ordinate was the second principal component (orthogonal principal component), expressed in *t*[1]O). *R* ^2^ *Y* represents the interpretability of the model; *Q* ^2^ represents the predictability of the model. Displacement test intercepts *R* ^2^ = 0.686 and *Q* ^2^ = 0.36 could well reflect the robustness of the model. The load diagram is about material for potential differences marked at both ends.

#### 3.5.2. OPLS-DA of VAWI Treatments

B-Q group versus B-H group OPLS-DA score chart was shown in Figure 3(f), displacement test (permutation test) diagram was shown in Figure 3(g), and OPLS-DA load diagram was shown in Figure 3(h).

### 3.6. The Decision Tree Model Analysis

Decision tree referred to the use of tree structure to represent the decision set, which was a kind of intuitive knowledge representation method, as well as efficient classifier [9]. The main ideas of constructing the decision tree were based on information theory for the tool; in all nonleaf nodes select important properties or property groups in all nonleaf nodes for superincumbent training set until meeting the termination conditions. The decision tree consisted of a root node, number of leaf nodes, and some nonleaf nodes. Root node is corresponding to the learning task. Each leaf node contained a classification. Decision tree was an important method of pattern recognition [10]. Its advantage is that rules are clear, with high classification accuracy.

Using R Weka toolkit's J48 algorithm (Java version of C4.5 algorithm) in the training of the decision tree, then set the minimum branch as more than two samples (http://www.cs.waikato.ac.nz/ml/weka/) [11].

Details were visible below, A-Q versus A-H and B-Q versus B-H.

#### 3.6.1. A-Q versus A-H

 See Figure 4.

#### 3.6.2. B-Q versus B-H

 See Figure 5.

### 3.7. Differential Protein Prediction

Swiss-Prot was used as the standard protein data in the database. We compared the SELDI data and amino acids molecular mass in our own software to discover the most similar proteins as prediction results. The detailed predicted information was listed in Tables 5 and 6.

## 4. Discussion

Pneumoconiosis is long-term inhalation of productive dust creative which is given priority to with pulmonary fibrosis of systemic disease and is one of the most serious occupational disease hazards in China. According to research, silicosis is the pathological changes of main pulmonary fibrosis [12]. The silicosis exact pathogenesis is still unknown, but a lot of evidence shows [13] alveolar macrophages, cytokines, Clara cells, oxidative stress, and the human's immunity to silica play an important role in silicosis occurrence and development. Because the pathogenesis of silicosis fibrosis is less known, treatment of silicosis has become a hot topic of current research [14]. In 1964, China government developed the western medicine aram si ping, then many other western medicines like piperaquine phosphate, hydroxy piperaquine phosphate, citric acid aluminum, and silicon, through a certain link in the process of pulmonary fibrosis to affect antifibrosis [15].

Tetrandrine [16] is resistant to silicosis of Chinese patent medicine; it influences collagen cross-linking reaction, inhibits collagen synthesis, and has inhibitory effect on the lungs lipids, prompting lipid to dissipate. Moreover, it acts on pulmonary vascular smooth muscle, removes vascular spasm, reduces vascular resistance, improves tissue perfusion, accelerates silicosis variable dissipated, and so on.

In recent years [17], silicosis treatment study from the molecular level is also increasing, such as TGF-*β* [18], as the key active factor of silicosis fibrosis, which can be induced by P38 splitting the original activating protease (mitogen activated protein kinase, MAPK) signaling pathway activation and exert its biological effect [6]. The block of TGF-*β* mediated P38 MAPK pathway was considered to be one of the important ways to inhibit the silicosis fibrosis.

There is much research on stem cell at present [19], including a lot of stem cells treatment research about a variety of diseases on clinic [20]. Ectomesenchymal stem cells (EMSC) were demonstrated in the treatment of pulmonary fibrosis disease in animal experiments. Stem cell and gene therapy jointly have been developed. MSC modified with hepatocyte growth factor gene recombinant adenovirus (Ad-HGF) was generated to EMSC. EMSC animal test showed that the curative of EMSC had better effect than MSC [21]. Our country also showed an MSC application in clinical treatment of silicosis. Although there are so many kinds of drugs in the treatment of silicosis, the overall effect is not agreeable [22]. All in all, it is of great urgency for us to test the curative effect of drugs listed.

Uygur classical medical recorded the* Vernonia anthelmintica* Willd functions such as cleaning abnormal phlegmatic temperament and abnormal black bile, spasmolysis, and relieving asthma and cough efficacy. This report was published in 1888.* Vernonia anthelmintica* Willd is a commonly used medicine. It is a typical medicinal resource in Xinjiang; the development of the medicine improves the economy of Xinjiang and people's living standard [10].

In 1998, sponsored by the ministry of health by the specialized research fund for the outstanding young scientific and technological personnel project “*Vernonia anthelmintica* Willd injection of preclinical studies,”* Vernonia anthelmintica* Willd injection was commonly used on the basis of uygur prescribed preparations through extracting sflavone and lactone content [23]. He carried on the strict pharmaceutical experiment [16], animal experiment, and clinical study, and the results showed that the preparation atomization inhibited the acute and chronic inflammation, restraining inflammation tissue PGE2, PGF1a synthesis, or release of inflammatory mediators. The asthma delayed-type hypersensitivities in peripheral blood, airway, and lung inflammatory cells infiltration were inhibited. The antigen against caused asthma symptoms was reduced. Antagonism of histamine and acetylcholine inflammation medium caused airway hyperresponsiveness and had obvious dosage-dependence manner. Clinical validation results showed that the treatment of asthma effective rate was 88.6%. In the end he developed the medicine successfully and applied for the patent. Subsequently, preliminary study revealed that VAWI inhibited rat pulmonary fibrosis [17, 18], indicating that VAWI could affect rat serums IL-8 and TNF-*β* and SOD and MDA and pulmonary fibrosis [24]. Proteomics technology includes purification technology, protein identification technology, and bioinformatics technology. The core technology of proteomics of comparative serum levels mainly contains the protein separation and identification technology. Mass spectrometry technology is the core of the protein identification technology. The most widely used technology at present is mainly the matrix assisted laser desorption ionization time of flight mass spectrometry (MALDI-TOF-MS) and electrospray ionization mass spectrometry (ESI-MS). On the basis of conventional mass spectrometry, to improve protein identification results, surface enhanced laser desorption ionization time of flight mass spectrometry (SELDI-TOF-MS) was developed.

This task group used Xinjiang specialty drug* Vernonia anthelmintica* Willd injection (VAWI) to treat patients with silicosis. Using series chips of different chemical surface modification can optionally be in combination with a set of proteins; by time of flight mass spectrometry detector, all proteins were determined in combination on the chip, and molecular weight was obtained by determination of flight time, following the analysis of protein profiles in different groups. This study via rapid high-throughput analysis of serum protein aimed to observe the VAWI curative effect for the treatment of silicosis fibrosis.

Taken together, our data demonstrated that there was obvious difference between silicosis patients before and after treatment in both foundation treatment group and VAWI treatment group. VAWI treatment in patients with silicosis fibrosis can lead to serum differences in protein expression. Further study will aim to identify the differential-expression proteins in the serum.

## Figures and Tables

**Figure 4:**
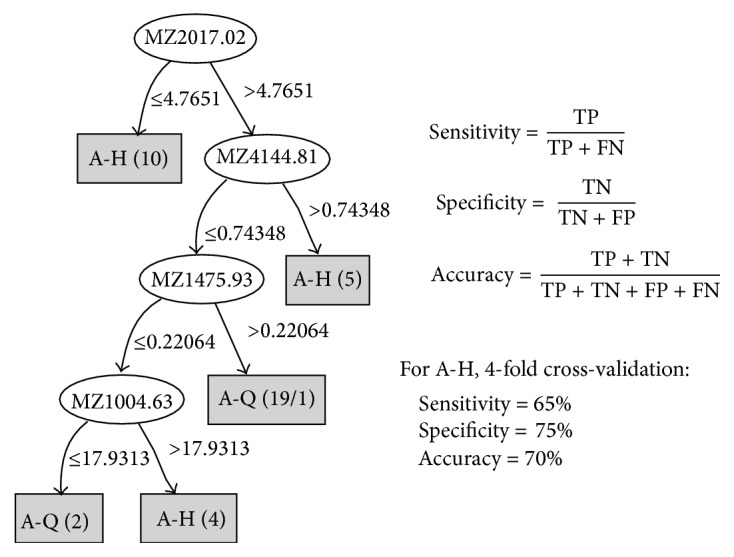


**Figure 5:**
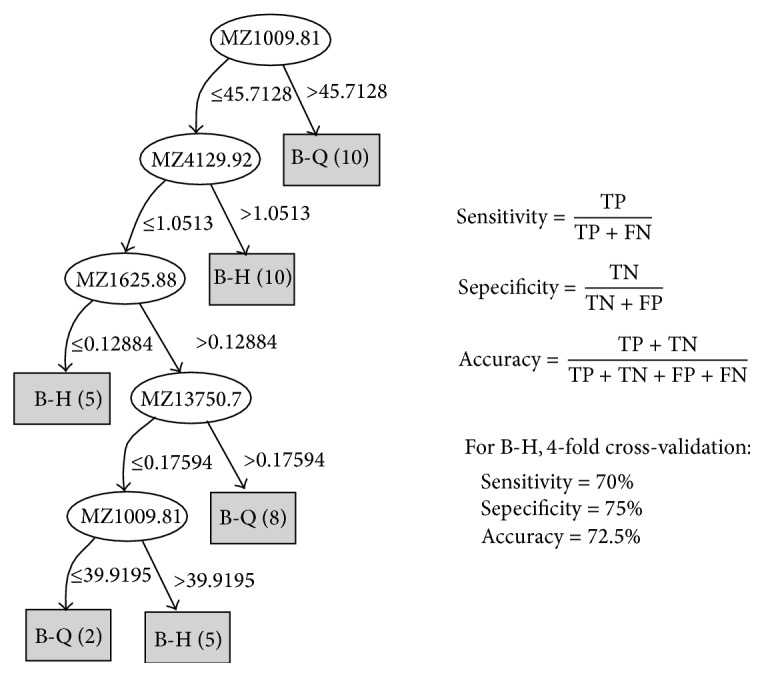


**Table 1:** Grouping and sample quantity in detail.

Groups	Group A-Q	Group A-H	Group B-Q	Group B-H
Clinical serum samples (cases)	20	20	20	20

**Table 2:** 

Model	Type	*A*	*N*	*R* ^2^ *X* (cum)	*R* ^2^ *Y* (cum)	*Q* ^2^ (cum)	Title
M1	OPLS-DA	1 + 1 + 0	40	0.278	0.803	0.132	A-Q versus A-H
M2	OPLS-DA	1 + 1 + 0	40	0.275	0.779	0.268	B-Q versus B-H

**Table 3:** A-Q versus A-H group differences in peak filtering.

SAMP_GRP	*p* value	*q* value	VIP	A-Q	A-Q	A-Q	A-Q	⋯	A-H	A-H	A-H	A-H	⋯
M1004_63	0.039989471	0.415818706	1.16467	22.95604025	22.75923654	26.75614087	19.74283745	?	19.86973828	22.78794232	18.45321078	15.93007727	?
M1019_45	0.002325058	0.220851981	2.23625	14.85695691	11.13177835	14.7041591	11.72356236	?	14.75674034	7.622252274	9.058499342	8.522960615	?
M1123_40	0.044054031	0.427081143	1.27539	4.612171108	3.101201241	2.334913184	4.269188349	?	3.197059112	4.448648643	0.312502018	0.26800485	?
M1146_35	0.036233902	0.40385461	2.52543	12.13544892	11.13549277	8.64778072	7.818968812	?	8.508263162	5.841696314	1.494565651	1.373904899	?
M1150_36	0.000707626	0.220851981	1.73778	11.59222276	5.959793927	9.678156541	7.526991361	?	6.925216398	4.861417086	2.548174863	1.98263985	?
M1207_98	0.013616562	0.299041303	2.71017	9.727282369	3.287925922	4.333702022	3.368115366	?	3.87734566	3.458762177	0.917583662	0.353833452	?
M1216_31	0.006389618	0.247157411	2.00268	9.068674912	10.40280222	14.98574501	12.96053724	?	13.06359903	6.247365256	4.877656968	3.084864891	?
?	?	?	?	?	?	?	?	?	?	?	?	?	?

**Table 4:** B-Q versus B-H group differences in peak filtering.

SAMP_GRP	*p* value	*q* value	VIP	B-Q	B-Q	B-Q	B-Q	⋯	B-H	B-H	B-H	B-H	⋯
M1004_63	0.036233902	0.117118797	4.47584	20.93159093	23.06330811	15.91894724	27.61736757	?	26.49269238	27.1344438	23.31498178	13.71342507	?
M1009_81	0.001432419	0.043568027	7.15663	48.79132299	46.95529883	48.87003438	54.19353556	?	43.63272086	43.98593065	44.75965014	42.28380538	?
M1234_44	0.026641846	0.101306267	1.27316	−0.796230455	1.453932033	3.966102148	4.890168124	?	4.084448225	−0.746040616	0.74709049	1.27757159	?
M1625_88	0.00271225	0.044951919	1.90781	0.786445814	2.048543355	2.910415223	9.575057215	?	3.389356609	−0.958622179	−0.105883517	1.318096198	?
M1982_50	0.048440933	0.135305336	1.00893	0.242692484	1.150477702	0.154858982	1.569664624	?	−1.489712488	−0.378626936	−1.455122429	1.014149656	?
M2506_71	0.029575348	0.106704052	1.86031	4.933817334	6.599231237	2.490269656	2.000045877	?	0.387148195	0.056803343	1.56353917	1.844580411	?
M2648_11	0.004859924	0.049028461	1.31311	0.569065076	−0.210226171	0.308796758	−0.226233975	?	1.755490095	2.811816812	0.19201706	0.346710763	?
?	?	?	?	?	?	?	?	?	?	?	?	?	?

**Table 5:** A-Q versus A-H group differential protein predicting outcomes.

SAMP_GRP	*p* value	*q* value	VIP	ID	MZ_SELDI	Predicted_protein	Theoretical_MZ	Gene_name
M2001_69	0.013616562	0.299041303	1.81131	M2001_69	2001.69	Amyloid beta a4 protein	1953.1	APP
M2017_02	0.00271225	0.220851981	2.50106	M2017_02	2017.02	Amyloid beta a4 protein	1953.1	APP
M4144_81	0.00365448	0.220851981	1.30244	M4144_81	4144.81	Plasma protease c1 inhibitor frag	4152.87	SERPING1
M4478_27	0.00365448	0.220851981	2.15012	M4478_27	4478.27	Alpha-1-antichymotrypsin, Alpha-1-antichymotrypsin frag	4343.65	SERPINA3
M4861_39	0.048440933	0.437642642	1.32359	M4861_39	4861.39	Neurosecretory protein vgf frag	4823.5	VGF
M5642_48	0.007295609	0.254483755	1.30207	M5642_48	5642.48	Fibrinogen alpha chain frag	5904.22	FGA
M7564_68	0.012079239	0.29154291	1.54656	M7564_68	7564.68	Osteopontin frag	7658.19	SPP1

**Table 6:** B-Q VS B-H group differential protein predicting outcomes.

SAMP_GRP	*p* value	*q* value	VIP	ID	MZ_SELDI	Predicted_protein	Theoretical_MZ	Gene_name
M1982_50	0.048440933	0.135305336	1.00893	M1982_50	1982.5	Amyloid beta a4 protein	1953.1	APP
M3164_50	0.039989471	0.122089598	1.30991	M3164_50	3164.5	Fibrinogen alpha chain frag	3262.47	FGA
M3379_28	0.000707626	0.043568027	1.5808	M3379_28	3379.28	Fibrinogen alpha chain frag	3262.47	FGA
M3539_61	0.021484375	0.091602781	1.04809	M3539_61	3539.61	Neutrophil defensin 1, neutrophil defensin 3	3448.09	DEFA1
M3825_21	0.000585556	0.043568027	1.10767	M3825_21	3825.21	Neurosecretory protein vgf frag	3688.03	VGF
M4062_14	0.021484375	0.091602781	1.21004	M4062_14	4062.14	Plasma protease c1 inhibitor frag	4152.87	SERPING1
M4076_27	0.023950577	0.095815237	1.32528	M4076_27	4076.27	Plasma protease c1 inhibitor frag	4152.87	SERPING1
M4129_92	0.013616562	0.073476016	1.06756	M4129_92	4129.92	Plasma protease c1 inhibitor frag	4152.87	SERPING1
M4144_81	0.00271225	0.044951919	1.16841	M4144_81	4144.81	Plasma protease c1 inhibitor frag	4152.87	SERPING1
M4161_30	0.005580902	0.051797761	1.08441	M4161_30	4161.3	Plasma protease c1 inhibitor frag	4152.87	SERPING1
M4257_41	0.004859924	0.049028461	1.3339	M4257_41	4257.41	Plasma protease c1 inhibitor frag	4152.87	SERPING1
M4421_61	0.007295609	0.058457352	1.1678	M4421_61	4421.61	Alpha-1-antichymotrypsin, alpha-1-antichymotrypsin frag	4343.65	SERPINA3
M4652_50	0.019233704	0.087243925	1.29353	M4652_50	4652.5	Neurosecretory protein vgf frag	4823.5	VGF
M4671_03	0.021484375	0.091602781	1.52031	M4671_03	4671.03	Neurosecretory protein vgf frag	4823.5	VGF
M4979_81	0.00365448	0.047243739	1.1581	M4979_81	4979.81	Neurosecretory protein vgf frag	4823.5	VGF
M6194_61	0.001209259	0.043568027	1.17401	M6194_61	6194.61	Apolipoprotein c-i	6432.35	APOC1
M6814_07	0.029575348	0.106704052	1.26252	M6814_07	6814.07	Transthyretin	6880	TTR
M7171_26	0.001689911	0.043568027	1.176	M7171_26	7171.26	Transthyretin	6880	TTR
M7765_40	0.010688782	0.067412421	2.95658	M7765_40	7765.4	Osteopontin frag	7658.19	SPP1
M7970_76	0.021484375	0.091602781	1.23426	M7970_76	7970.76	Osteopontin frag	7658.19	SPP1
M8033_66	0.019233704	0.087243925	1.54665	M8033_66	8033.66	Osteopontin frag	7658.19	SPP1
M8139_51	0.039989471	0.122089598	1.34496	M8139_51	8139.51	Hemoglobin subunit beta	7931.8	HBB
M9187_15	0.026641846	0.101306267	3.63548	M9187_15	9187.15	Apolipoprotein a-ii	9303.65	APOA2
M9285_47	0.017181396	0.082753919	2.37145	M9285_47	9285.47	Apolipoprotein a-ii	9303.65	APOA2
M9414_68	0.036233902	0.117118797	2.14727	M9414_68	9414.68	Apolipoprotein a-ii	9303.65	APOA2
M9440_33	0.029575348	0.106704052	1.58489	M9440_33	9440.33	Apolipoprotein a-ii	9303.65	APOA2
M9492_65	0.002325058	0.044562279	1.54296	M9492_65	9492.65	Apolipoprotein a-ii	9303.65	APOA2
M13750_7	0.00365448	0.047243739	1.49453	M13750_7	13750.7	Cystatin-c	13347.14	CST3
M13886_7	0.004859924	0.049028461	1.84674	M13886_7	13886.7	Cystatin-c	13347.14	CST3
M14042_5	0.003152847	0.046165766	1.66163	M14042_5	14042.5	Transthyretin	13761.41	TTR
M15118_8	0.010688782	0.067412421	1.14482	M15118_8	15118.8	Hemoglobin subunit beta	15867.22	HBB
M15858_3	0.036233902	0.117118797	1.09762	M15858_3	15858.3	Hemoglobin subunit beta	15867.22	HBB
M16052_7	0.00271225	0.044951919	2.55714	M16052_7	16052.7	Hemoglobin subunit beta	15867.22	HBB
M16231_2	0.008308411	0.061599564	1.65407	M16231_2	16231.2	Hemoglobin subunit beta	15867.22	HBB
